# Effect of improvement measures in reducing interruptions in a Japanese hospital pharmacy using a synthetic approach based on resilience engineering and systems thinking

**DOI:** 10.1186/s12913-023-09346-2

**Published:** 2023-04-03

**Authors:** Takahiro Kojima, Noriyasu Kinoshita, Harumi Kitamura, Koji Tanaka, Ayumi Tokunaga, Satoshi Nakagawa, Takeru Abe, Kazue Nakajima

**Affiliations:** 1 Department of Clinical Quality Management, Osaka University Hospital, 2-15 Yamadaoka, Suita, Osaka 565-0871 Japan; 2 Department of Orthopedic Surgery, Graduate School of Medicine, Osaka University, 2-2 Yamadaoka, Suita, Osaka 565-0871 Japan; 3 Osaka A&M Law office, 7F Kitahama Exel Building, 2-6-11, Kitahama, Chuo-Ku, Osaka, 541-0041 Japan; 4 Department of Pharmacy, Osaka University Hospital, 2-15 Yamadaoka, Suita, Osaka 565-0871 Japan; 5 Department of Gastroenterological Surgery, Graduate School of Medicine, Osaka University, 2-2 Yamadaoka, Suita, Osaka 565-0871 Japan; 6 Department of Obstetrics and Gynecology, Graduate School of Medicine, Osaka University, 2-2 Yamadaoka, Suita, Osaka 565-0871 Japan; 7 Department of Quality and Safety in Healthcare, Yokohama City University Medical Center, 4-57, Urafune-Cho, Minami-Ku, Yokohama, Kanagawa 232-0024 Japan; 8 Advanced Critical Care and Emergency Center, Yokohama City University Medical Center, 4-57, Urafune-Cho, Minami-Ku, Yokohama, Kanagawa 232-0024 Japan

**Keywords:** Interruptions, Hospital pharmacy, Patient safety, Performance adjustment, Adaptive behavior, Resilience engineering, Systems thinking, Synthetic approach, Systemic problem, Safety-II

## Abstract

**Background:**

Workflow interruptions in pharmacies contribute to dispensing errors, a high-priority issue in patient safety, but have rarely been studied from a systemic perspective partly because of the limitations of the conventional reductionistic approach. This study aims to identify a mechanism for the occurrence of interruptions in a hospital pharmacy and find interventional points using a synthetic approach based on resilience engineering and systems thinking, and assess implemented measures for reducing them.

**Methods:**

At a Japanese university hospital, we gathered information about performance adjustments of pharmacists in the inpatient medication dispensing unit for oral and topical medicines (IMDU-OT) and nurses in the inpatient wards (IPWs) in the medication dispensing and delivery process. Data about the workload and workforce of pharmacists were collected from hospital information systems. Telephone inquiries and counter services in the IMDU-OT, the primary sources of interruptions to pharmacists' work, were documented. The feedback structure between the IMDU-OT and the IPWs was analyzed using a causal loop diagram to identify interventional points. The numbers of telephone calls and counter services were measured cross-sectionally before (February 2017) and four months after implementing measures (July 2020).

**Results:**

This study found that interruptions are a systemic problem emerging from the adaptive behavior of pharmacists and nurses to their work constraints, such as short staffing of pharmacists, which limited the frequency of medication deliveries to IPWs, and lack of information about the medication dispensing status for nurses. Measures for mitigating cross-system performance adjustments—a medication dispensing tracking system for nurses, request-based extra medication delivery, and pass boxes for earlier pick-up of medicines—were introduced. Following their implementation, the daily median number of telephone calls and counter services was significantly reduced (43 to 18 and 55 to 15, respectively), resulting in a 60% reduction in the total number of interruptions.

**Conclusion:**

This study found interruptions in the hospital pharmacy as a systemic problem that can be reduced by mitigating difficulties being compensated for by clinicians' cross-system performance adjustments. Our findings suggest that a synthetic approach can be effective for solving complex problems and have implications for methodological guidance for Safety-II in practice.

## Introduction

Work interruptions and distractions are significant contributors to dispensing errors in hospital pharmacies, along with high workloads, low staffing levels, mix-up of look-alike/sound-alike drugs, lack of experience, and urgent deadlines/hurrying of tasks [1]. A high frequency of interruptions has been reported, ranging from every 2 to 6 min [2–4], making this a high-priority issue in patient safety. Telephone calls are the prominent interruption sources for inpatient pharmacies [2–4], and nurse inquiries about the prescription status accounting for a substantial part of the interruptions [5]. Human behaviors that might produce interruptions have been rarely studied, partly because conventional patient safety, reacting to incidents, and using a reductionistic approach and a linear causal model, fail to capture the dynamic nature of human behavior in adapting to given conditions to accomplish work [6–9]. Recognition of healthcare systems as complex adaptive systems has attracted the interest of healthcare providers and researchers toward a synthetic approach to solving complex problems [10, 11]. Both resilience engineering and systems thinking are theories for a synthetic approach to understanding systems’ behavior emerging from human interactions. Resilience engineering, a new paradigm in safety management, provides a perspective on how people work adaptively through performance adjustments in an environment with changes and constraints to achieve their goals [12]. Systems thinking helps develop a causal model of systems’ interactions with feedback loops [13]. However, there is no study about how complex problems such as interruptions in health care systems can be solved using a synthetic approach.

Through daily patient safety activities, we recognized that pharmacists in our university hospital experienced frequent interruptions during their dispensing work for inpatients owing to telephone calls from nurses in inpatient wards and counter services for urgent provisions of dispensed medicines to staff in the inpatient ward. This study aimed to identify a mechanism for these kinds of interruptions in a hospital pharmacy to find improvement measures using a synthetic approach and assess the effect of the implemented measures in reducing interruptions.

## Methods

### Study setting

The study setting was the inpatient medication dispensing unit for oral and topical medicines (IMDU-OT) in the Pharmacy Department of Osaka University Hospital (OUH) (1,086 beds) in Japan. The workforce in the IMDU-OT has been insufficient because many pharmacists have become engaged in medication guidance for inpatients and mixing anticancer drugs, which were incentivized by the national payment scheme. Medication dispensing processes in the IMDU-OT comprise three major steps: 1) retrieval of the prescription data from electronic patient record systems, checking of prescriptions, and preparation of the prescribed medicines (preparations); 2) independent checks of prepared medications (independent double-checks); 3) setup of scheduled deliveries (deliveries). Five or six pharmacists operated the IMDU-OT during the day shift, including only a few experienced pharmacists eligible for double-checking. Owing to regulations, pharmacy technicians or assistants were unavailable in Japanese pharmacies until April 2020.

Dispensed oral and topical medicines are delivered to 24 inpatient wards using automatic transport equipment four times per day at 10:00, 14:00, 16:00, and 20:00. When dispensed medicines are needed earlier than regular delivery times, nurses from the inpatient wards call the IMDU-OT and pick up the medications at the service counter in the IMDU-OT. The IMDU-OT receives telephone inquiries related to the dispensing processes of oral and topical medicines as well as injectables from all hospital areas and provides counter services for all urgently needed medicines for inpatients. Pharmacists in the IMDU-OT must respond to phone calls and counter services, even during dispensing processes.

### Targeted problems

When the Department of Clinical Quality Management (DCQM) of OUH interviewed several pharmacists about hazardous factors for medication dispensing incidents as part of its daily safety activities, the chronic problems of frequent workflow interruptions and low staffing in the IMDU-OT were recognized. Pharmacists indicated that the predominant causes of interruptions were telephone inquiries from nurses in inpatient wards and their counter services in the IMDU-OT for urgently providing dispensed medicines to staff in inpatient wards. The pharmacy department had asked nurses in inpatient wards to reduce calls, but the situation never improved.

To reduce interruptions during dispensing of medicines due to telephone inquiries and counter services for inpatients, the DCQM convened the project team that comprised healthcare professionals involved in the medication processes and patient safety, including physicians, nurses, pharmacist leaders in the IMDU and other units, the director and vice directors of the pharmacy department, and the DCQM's multi-professional members. According to a suggestion from pharmacists of the project team, the operational process for oral and topical medicines was selected as an improvement target rather than injectables because the dispensing and delivery system of injectables for inpatients was more complicated due to the involvement of an outsourcing contractor and having different operational rules. The project was launched to reduce interruptions due to telephone inquiries and counter services related to dispensing oral and topical medicines for inpatients.

### Study phases and their components

This project had two phases. In phase 1, which started in February 2017, we captured the performance adjustments of pharmacists in the IMDU-OT and nurses in inpatient wards in medication dispensing and delivery processes, identified a mechanism for the occurrence of interruptions and interventional points, and developed an interventional scheme for improvement. In phase 2, we implemented the intervention in daily operations and assessed the outcome of the changed practice.

### Phase 1

#### Capturing performance adjustments in everyday clinical work

Based on the analysis principle of "breadth-before-depth" in resilience engineering [12], we examined how the limited number of pharmacists in the IMDU-OT (one system) adjusted their performance in medication dispensing and delivery processes to provide medications to inpatients on time as per physician orders, focusing on interaction with nurses in inpatient wards (the other system).

To collect information on performance adjustments of pharmacists and nurses and their reasons, we interviewed several pharmacists, including the project members, observing their work in the IMDU-OT. Questions included how to accomplish dispensing work under limited workforce and time and the frequency and reasons for telephone inquiries and counter services. The interview with pharmacists indicated staffing support for dispensing from other units, the limited frequency of medication deliveries to inpatient wards, little information about individual performance adjustments, and no recording system for telephone inquiries and counter services. We extracted workload data related to medication dispensing from the hospital information systems and obtained the workforce data of pharmacists from their work schedules. Workload data included the number of prescriptions dispensed and the number of recipes in the prescriptions independently double-checked by each pharmacist from January 1, 2017, to December 31, 2017.

The data on the frequency and contents of phone calls received and pick-up counter services provided in the IMDU-OT were recorded by the DCQM's pharmacist using a structured sheet, who responded to all of them during business hours of the five weekdays from February 21 to 27, 2017. In addition, we also asked nurses of the project members to provide context for their behavior regarding telephone inquiries and the use of pick-up services.

#### Identifying a mechanism for interruptions and interventional points

We analyzed the interactions between the two systems of the IMDU-OT and inpatient wards using a causal loop diagram, a qualitative tool used in systems thinking to visualize feedback among systems [13]. Drawing a causal loop diagram requires information about gaps between desired and actual conditions in the system and human or systems behaviors adjusted to such gaps, which were obtained in the first component of phase 1. The prepared diagram was used to identify a mechanism for the emergence of a systemic problem through interactions between different systems and interventional points to interdependent systems for a fundamental solution.

#### Developing an intervention scheme in a synthetic manner

The project team developed an intervention scheme based on a safety management strategy in resilience engineering where performance adjustment should be managed and dampened if it looks like it is going in the wrong direction [12]. Specific measures to mitigate performance adjustments across systems between the IMDU-OT and inpatient wards identified in the causal loop diagram were examined. We adopted a policy that the intervention would not increase the workload of pharmacists in the IMDU-OT, nor create an additional burden for nurses and staff in inpatient wards.

### Phase 2

#### Implementing the intervention in daily operations

Based on the findings of phase 1, the project team proposed three improvement measures for reducing interruptions in the IMDU-OT to the hospital patient safety committee and obtained permission for implementation.

#### Assessing the outcome of changed practices

A before-and-after study design was used to measure the overall effect of the intervention on interruptions in the IMDU-OT. Two outcome indicators were measured: the number of telephone inquiries about oral and topical medicines by their reasons and the number of counter services by type of medicine. They were measured during phase 1 (February 21 to 27, 2017) and four months after implementing all three components using the data documented by pharmacists for five consecutive weekdays (July 27 to 31, 2020). During the same period, utilization data of the medication dispensing tracking system and the extra delivery system were extracted from the hospital information systems, and those of pass boxes were extracted from telephone request records.

### Statistical Analysis

In phase 1, the box and whisker plots were used to display variation in the number of recipes independently double-checked by each pharmacist per day. In phase 2, Mann–Whitney U test was used to compare the differences in the number of telephone inquiries about oral and topical medicines and the number of counter services before and after the intervention. A two-sided *P* value less than 0.05 was considered statistically significant. We utilized the false discovery rate (FDR) control method and set 5% FDR [14], which would be acceptable against potential false positives, as addressing an issue of multiple tests when comparing for contents of telephone inquiries and counter services. Data were analyzed with JMP® Pro 15.0 for Windows (SAS Institute Inc., Cary, NC, USA).

## Results

### Phase 1

#### Performance adjustments of pharmacists and nurses

**Fig. 1:**
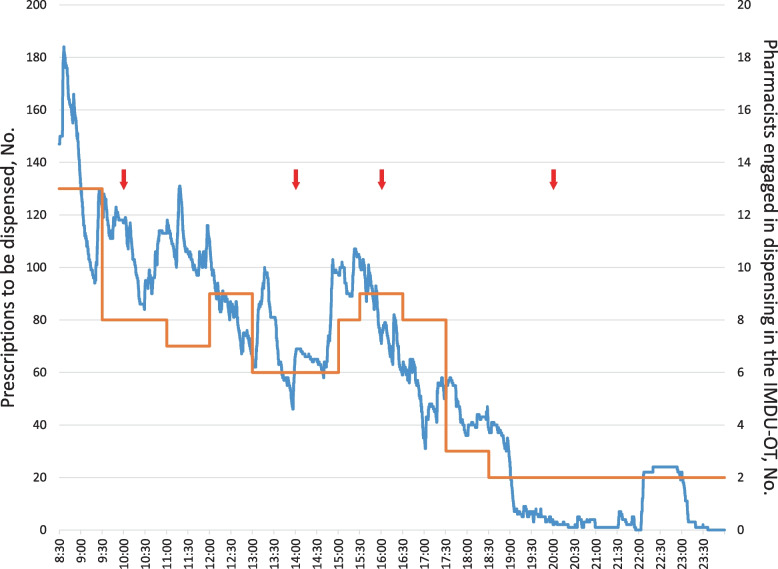
Number of prescriptions to be dispensed and pharmacists engaged in dispensing. Blue lines indicate the number of prescriptions to be dispensed; red lines, the number of pharmacists engaged in dispensing medicines; red arrows, the delivery time

In the project’s first phase, we identified three types of performance adjustments for the timely completion of medication dispensing by the limited workforce in the pharmacy department. Figure 1 shows that more pharmacists were engaged in dispensing medicines of prescriptions (up to 13 pharmacists) than those assigned in the IMDU-OT (6 pharmacists) to dispense 999 prescriptions. The pharmacy department had routinely adopted the staffing support system for the IMDU-OT from its other units, which was scheduled in advance, considering workload concentrations in the IMDU-OT.

**Fig. 2:**
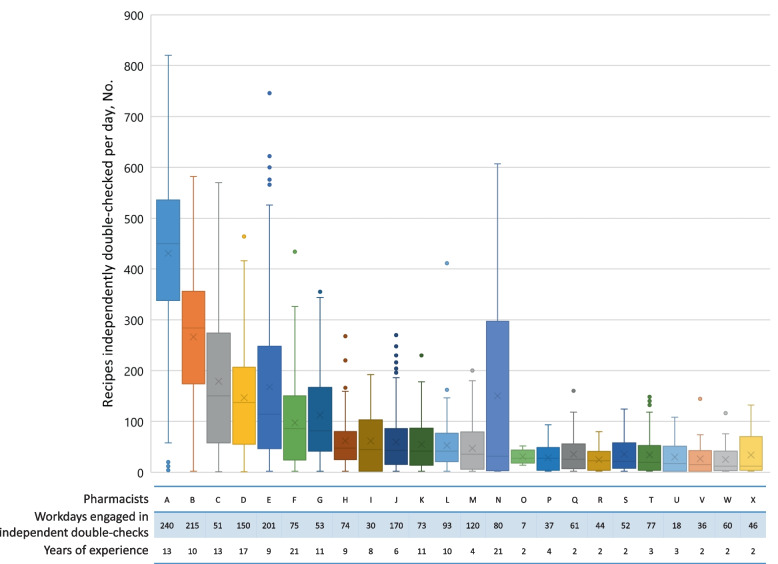
Distribution of daily recipes about oral and topical medicines independently double-checked by the pharmacist. Boxes indicate the IQR; dots, outliers; line within the box, median; crosses within the box, mean; the end of the lower whisker, minimum; the end of the upper whisker, maximum

Figure 2 shows that the number of double-checked recipes for oral and topical medicines varied among pharmacists. They were mainly categorized into two groups: one with higher medians, larger interquartile ranges (IQRs), and longer upper whiskers with greater highest values or outliers (pharmacists A, B, C, D, E, F, G, N) and the other with lower medians, smaller IQRs and shorter upper whiskers with smaller highest values or outliers (the rest of the pharmacists). Specific pharmacists with longer clinical experiences, including pharmacists A, B, and N, who were a leader of the IMDU and subleaders of the IMDU-OT and IMDU for injections, respectively, conducted larger volumes of double-checks according to their workload. The pharmacy department also determined the frequency of scheduled medication deliveries only four times a day because medication delivery-related tasks required pharmacists’ workforce.

Table 1 shows the total number of telephone inquiries and counter services handled in the IMDU-OT during business hours for five consecutive days. The overall telephone calls received in the IMDU-OT was an average of 139 per day-shift (103 from inpatient wards, consisting of 42 for oral or topical medicines and 61 for injections, 31 from outpatient departments, and 5 from others). The main reasons for nurses’ phone calls to the IMDU-OT were requests for earlier dispensing and use of counter services for earlier receipts (58%), inquiries about medication dispensing and delivery status (25%), and requests for earlier-than-scheduled delivery using automatic transport equipment (9%). The total number of counter services was 49 times per day-shift (13 for oral or topical medicines, 19 for injectables, and 17 for others). Pharmacists were interrupted by answering phone calls every 3.5 min, providing counter services every 9.8 min, and both together every 2.6 min.

**Table 1:** Frequencies of and reasons for telephone inquiries received in the IMDU-OT during business hours

	Inpatient wards				Outpatient departments		Others		Total	
	Oral or topical medicines		Injectables							
**Reasons for inquiries**	N	(%)	N	(%)	N	(%)	N	(%)	N	(%)
Requests for earlier receipts at the service counter	121	(57.9%)	155	(50.5%)	12	(7.6%)	1	(4.2%)	289	(41.5%)
Checking the dispensing and delivery status	53	(25.4%)	45	(14.7%)	3	(1.9%)	0	(0%)	101	(14.5%)
Requests for earlier-than-scheduled deliveries with machine transportation	19	(9.1%)	90	(29.3%)	134	(85.4%)	1	(4.2%)	244	(35.0%)
Others	16	(7.7%)	17	(5.5%)	8	(5.1%)	22	(91.6%)	63	(9.0%)
Total phone calls for five consecutive days	209	(100%)	307	(100%)	157	(100%)	24	(100%)	697	(100%)
Average phone calls per day	42		61		31		5		139	

Telephone requests to pick up dispensed medicines at the service counter instead of waiting for regular or earlier-than-scheduled deliveries inferred that the current frequency of regular machine-deliveries was insufficient for nurses to act on physicians' medication orders. Nurse' telephone inquiries about the dispensing and delivery status indicated their need to ascertain dispensing and delivery statuses but no ways to track them. Interviews with nurses found such needs that nurses wanted to know whether intended medications were under dispensing and delivery or had been delivered to their wards and processed by other nurses when they did not find their patients' medicines in a specific place in the nurse station. These data showed that nurses' telephone calls and use of counter services were their performance adjustments to accomplish their work in the limited regular medication deliveries and the lack of information about dispensing and delivery status.

#### Mechanism for the emergence of interruptions

**Fig. 3:**
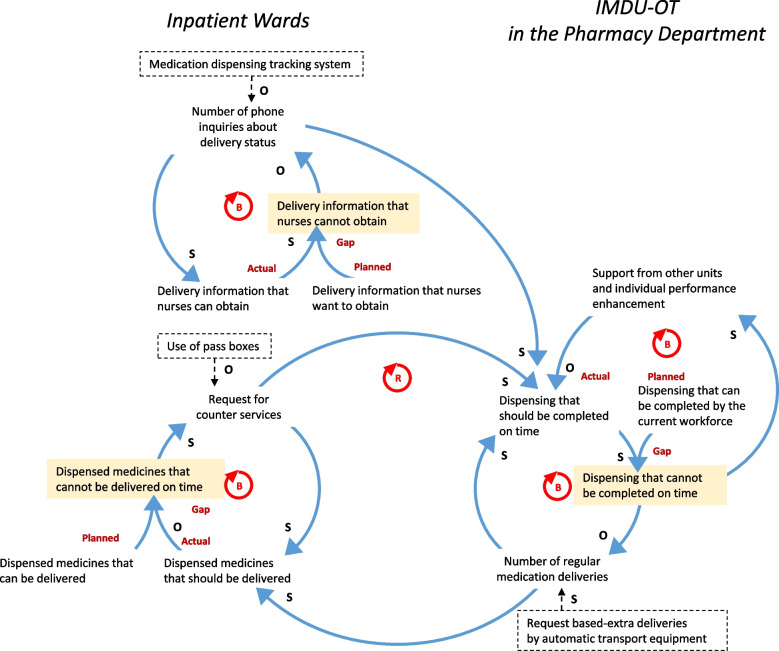
Feedback structure between the IMDU-OT and the inpatient wards. S indicates that one variable moves in the same direction as the other variable; O indicates that one variable moves in the opposite direction; B, a balancing loop; R, a reinforcing loop; a dotted squire box, a component of the intervention

The causal loop diagram shows the interactions of adaptive behavior of pharmacists in the IMDU-OT and nurses in inpatient wards (Fig. 3). To fill the gap between the actual workload that should be completed and the workload that the current workforce can complete, the pharmacy department employed departmental staffing support, individual performance enhancement, and limited medication deliveries. In inpatient wards, the pharmacists’ cross-boundary tactic caused the gap between the number of medicines that should be delivered and the number of medicines that are regularly delivered. To address this gap, nurses telephoned the IMDU-OT to obtain necessary medicines at the service counter. The lack of information about medication dispensing and delivery statuses for nurses also created the gap between the number of medicines whose locations they wanted to know and the number of medicines whose locations they knew. Such adaptive behavior of nurses caused frequent interruptions for pharmacists’ work through phone calls and counter services.

#### Determining the interventional components

Three improvement measures were adopted to mitigate performance adjustments across the boundaries of the two different systems for reducing interruptions in the IMDU-OT. The first measure was the use of pass boxes for both oral and topical medicines and injectables for providing medicines to the staff of inpatient wards instead of pharmacists' counter services in the IMDU-OT. The second one was the introduction of the medication dispensing tracking system for oral and topical medicines integrated with electronic patient record systems without telephonic queries. The system displays status messages for every prescription of each patient, namely "currently dispensing," "dispensed," "sent to ward," and "in pass box." The third was request-based extra deliveries of dispensed medicines using automatic transport equipment other than the scheduled deliveries. When a nurse sends an empty container for the automatic transport equipment located in each inpatient ward to the IMDU-OT between 10:00 and 16:00 on weekdays without a telephone request, all medications dispensed by that time are delivered to the requesting ward.

### Phase 2

#### Implementation of the interventional measures

The use of pass boxes started on May 15, 2017. The medication dispensing tracking system for nurses in inpatient wards and request-based extra medication deliveries were implemented together on March 16, 2020, after the hospital approved the budget for the medication dispensing tracking system and hired a clerk for the pharmacy department for tasks related to the extra delivery system.

#### Effect of the implemented measures

Regarding the utilization of the interventional components, the median access [IQR] to the medication dispensing tracking system was 75 [51–81] times per day, ranging from 2 to 46 times in each inpatient ward (11 times in the median). Extra deliveries of dispensed medicines were requested 25 [24-28] times per day, ranging from 1 to 20 times in each inpatient ward (4 times). Pass boxes were used 18 [18-23] times per day.

Table 2 shows that following the intervention, the total number of telephone inquiries related to oral and topical medicines in the median [IQR] decreased significantly (from 43 [38–46] to 18 [18, 19] times per day, *p* = 0.032). For types of inquiries, a significant reduction was observed in checking medication dispensing and delivery statuses (from 10 [9-12] to 1 [1], *p* = 0.008). Requests for earlier dispensing and receiving at the service counter tended to be reduced but not significantly (from 26 [22-28] to 9 [9-13], *p* = 0.032). The requests for an earlier-than-scheduled delivery with machine transportation were not significantly decreased. The total number of counter services decreased significantly (from 55 [51–56] to 15 [15] times per day, *p* = 0.008). Counter services were used only for medicines to be stored in inpatient wards or narcotics, or when all pass boxes were occupied. The total interruptions due to answering phone calls and providing counter services decreased from every 2.6 to 6.7 min.

**Table 2:** Change in daily telephone inquiries and counter services by the implemented measures

	Before	After		
Sources of interruptions	Median (IQR)	Median (IQR)	*P* Value	FDR-adjusted P Value
Telephone inquiries				
Total	43 (38–46)	18 (18–19)	.032	
Requests for earlier receipts at the service counter	26 (22–28)	9 (9–13)		.032
Checking the dispensing and delivery status	10 (9–12)	1 (1–1)		.008^a^
Requests for earlier-than-scheduled deliveries with machine transportation	4 (3–4)	1 (1–3)		.151
Others	3 (3–4)	4 (4–6)		.548
Counter services				
Total	55 (51–56)	15 (15–15)	.008	
Topical and oral medicines	19 (18–22)	2 (2–3)		.008^b^
Injectable medicines	14 (12–14)	1 (1–2)		.008^b^
Others	17 (15–19)	12 (12–13)		.095

^a,b ^denote statistical significance at *P*<.013 and *P*<.017, respectively

## Discussion

This study found that interruptions to pharmacists' work due to telephone inquiries and counter services were a systemic problem that emerged from the adaptive behavior of pharmacists and nurses to the work constraints such as short staffing, limited frequency of medication deliveries, and lack of information about the medication dispensing status. The measures for mitigating cross-system performance adjustments—specifically, the medication dispensing tracking system, additional medication deliveries, and the use of pass boxes—reduced interruptions by 60%. The information flow (of the dispensing status), the rules (for obtaining medicines earlier), and the feedback loops (between the two systems) that we intervened have been effective leverage points to change the system’s behavior [15]. The medication dispensing tracking system reduces interruptions by nurses’ phone calls in a hospital pharmacy [16].

The strengths of this study are as follows. First, to the best of our knowledge, this is the first study to determine how interruptions in a hospital pharmacy were caused and mitigated from a systemic perspective. Second, this study demonstrates the methodological guidance for implementing the theories of a synthetic approach into safety management practices. In practicing Safety-II based on resilience engineering, concrete ways of learning from everyday clinical work, analytical tools for identifying systemic problems, and the scientific evidence for its effectiveness have been challenging [17]. We illustrated how to learn from work-as-done where performance is continually being adjusted, to analyze the non-linear effect of interactions between the different systems with a dynamic thinking tool, and to identify effective measures for reconciling work-as-imagined, defined as work that should be performed according to plans or rules, and work-as-done [12].

The study results illuminate hidden risks of clinicians’ performance adjustments that enable the law of stretched systems and the law of fluency [18]. Among the three adaptive tactics for extending the capacity for maneuver in the IMDU-OT, extending the capacities of individual pharmacists was reported as a first-order problem solving to increasing demand [19] and has a possibility of falling into unacceptable performance [20], such as the criminal case of a medication error [21]. Creating constraints to other systems, which is a typical pattern of adapting to the shortfall in capacity for maneuver [22], may cause additional problems in the interrelated systems through a mechanism of the fallacy of composition or a problematic pattern of systems behavior called “shifting the burden” [23, 24]. The study indicates that solving a systemic problem requires looking into interrelated systems widely, not a single system locally. Reciprocity, which benefits both pharmacists and nurses, is also essential in determining improvement measures to extend the adaptive capacity of interconnected systems [25, 26].

Our study had several limitations. First, the study is conducted in a single Japanese university hospital, and so may limit the generalizability of the study findings. Yet the literature found commonalities in causes or sources of interruptions in other hospital pharmacies with ours [2–5]. A more important aspect of this study is the process of identifying a systemic problem and effective improvement measures unique to the context using a synthetic approach. Second, the daily prescription shown in Fig. 1 may not represent the regular working situation in the IMDU-OT because it was based on work performance data from one day in August 2017. This could limit the generalizability of the study findings. However, the number of prescriptions handled that day (999) was within the normal range (within mean plus 2 standard deviations, SD), considering the prescription data from all business days in 2017 [daily mean (SD): 859 (94)]. In addition, the staffing support scheme for the IMDU-OT on that day followed a typical patterns based on the time and day of the week. Thus, those would minimize this limitation. Third, the study targeted interruptions regarding oral and topical medicines only, leaving other significant sources of interruptions to pharmacists untouched (e.g., telephone inquiries about injectables for inpatient wards and novice pharmacists’ requests for guidance from skilled pharmacists). Fourth, the study did not assess the long-term effect of the intervention because data collection over time became unperformable due to the deteriorating situation of the COVID-19 pandemic. As of now, at least, the utilization data of the machinery extra delivery system has been at a similar level as the immediate post-intervention, and telephone inquiries about the dispensing status have remained a few, according to pharmacists' recognition.

## Conclusions

Our study found that interruptions in the hospital pharmacy due to telephone inquiries by nurses and their use of counter services are a systemic problem that can be reduced by mitigating work-related difficulties that are compensated for by human performance adjustments. It also showed that a synthetic approach to safety management can be useful in finding a systemic problem threatening patient safety and effective interventional points in complex adaptive systems. Our findings have implications for patient safety practices and regulations. Healthcare organizations and patient safety experts need to develop proactive safety management in dynamic work environments, gaining insight into the risks of adversity behind the fluent performance. Healthcare regulators also should promote such practices and research, which have been introduced into health policies in some countries [27, 28]. Further studies are required to validate the effectiveness of a synthetic approach to solving interruptions and other systemic problems in different settings.

## Data Availability

The data that support the findings of this study are not publicly available due to containing potentially identifying information of health care workers in the studied hospital and are available from the authors upon reasonable request and with permission of the hospital.

## Abbreviations

DCQM: Department of Clinical Quality Management
FDR: False discovery rate
IMDU-OT: Inpatient medication dispensing unit for oral and topical medicines
IPWs: Inpatient wards
IQRs: Interquartile ranges
OUH: Osaka University Hospital
